# Supplementary material for: The Effect of Extremely Low-Frequency Electromagnetic Fields on Inflammation and Performance-Related Indices in Trained Athletes: A Double-Blinded Crossover Study
Source: Int J Mol Sci. 2023 Aug 30;24(17):13463. doi: 10.3390/ijms241713463 (PMC10487818; doi:10.3390/ijms241713463)
Supplement: Supplementary file 1 [file ijms-24-13463-s001.zip › Supplementary S1.pdf]

	Cluster 1<br>n = 4	Cluster 2<br>n = 5	*p* value
Age (y)	21.7 ± 3.3	32.4 ± 1.3	**0.016**
Weight (kg)	64.4 ± 4.9	73.9 ± 7.2	0.111
BMI (kg/m^2)	20.4 ± 1.34	23.5 ± 2.35	0.063
Vo2peak (ml/kg/min)	60.2 ± 4.4	55.0 ± 7.9	0.413
Fat mass (kg)	6.7 ± 2.5	11.6 ± 1.37	**0.032**

All data presented as mean and S.D.
