# Supplementary material for: The Effect of Extremely Low-Frequency Electromagnetic Fields on Inflammation and Performance-Related Indices in Trained Athletes: A Double-Blinded Crossover Study
Source: Int J Mol Sci. 2023 Aug 30;24(17):13463. doi: 10.3390/ijms241713463 (PMC10487818; doi:10.3390/ijms241713463)
Supplement: Supplementary file 1 [file ijms-24-13463-s001.zip › Supplementary S2.pdf]

ROF

		Cluster 1	Cluster 2	P value
**Active**	BL	3±1	1±1	0.137
	60P	3±1	2±1	0.192
	24P	3±1	2±3	0.706
**Non-Active**	BL	2±1	1±2	0.579
	60P	2±1	3±2	0.842
	24P	4±1	2±2	0.109

VAS

		Cluster 1	Cluster 2	P value
**Active**	BL	5.4±4.2	3.5±3.4	0.433
	60P	8.7±3.3	4.6±4.1	0.096
	24P	5.4±4.1	4.4±3.9	0.692
**Non-Active**	BL	2.9±2.7	2.5±2.5	0.889
	60P	5.7±2.9	2.3±2.3	0.201
	24P	4.8±2.4	3.0±3.5	0.488

Rate of fatigue (ROF), Visual analog scale (VAS). All data presented as mean and S.D.
